# Prognostic Value of Glioma Cancer Stem Cell Isolation in Survival of Primary Glioblastoma Patients

**DOI:** 10.1155/2014/838950

**Published:** 2014-12-11

**Authors:** Byung Ho Kong, Ju Hyung Moon, Yong-Min Huh, Jin-Kyoung Shim, Ji-Hyun Lee, Eui-Hyun Kim, Jong Hee Chang, Dong-Seok Kim, Yong-Kil Hong, Sun Ho Kim, Su-Jae Lee, Seok-Gu Kang

**Affiliations:** ^1^Department of Medical Science, The Catholic University of Korea College of Medicine, 222 Banpo-daero, Seocho-gu, Seoul 137-701, Republic of Korea; ^2^Department of Neurosurgery, Severance Hospital, Yonsei University College of Medicine, 50-1 Yonsei-ro, Seodaemun-gu, Seoul 120-752, Republic of Korea; ^3^Department of Radiology, Severance Hospital, Yonsei University College of Medicine, 50-1 Yonsei-ro, Seodaemun-gu, Seoul 120-752, Republic of Korea; ^4^Department of Neurosurgery, Seoul St. Mary's Hospital, The Catholic University of Korea College of Medicine, 222 Banpo-daero, Seocho-gu, Seoul 137-701, Republic of Korea; ^5^Department of Life Science, Hanyang University, 17 Haendang-dong, Seongdong-gu, Seoul 133-791, Republic of Korea

## Abstract

Cancer stem cells (CSCs) have been reported to be critical in the initiation, maintenance, and progression of cancers. The expression of stem cell markers, such as podoplanin (PDPN), CD133, and nestin, may have been correlated with malignant progression. However, the effects of CSCs and stem cell markers on clinical outcomes in cancer patients remain unclear. In this study, we assessed the prognostic roles of glioma CSCs (gCSCs) isolation and stem cell markers in patients with primary glioblastoma (pGBM). A cohort of 39 patients with pGBM was separated into two groups, those positive or negative for gCSCs, and the correlation between gCSC and patient survival was evaluated. We observed significantly different cumulative survival (*P* = 0.045) when comparing patients positive for gCSCs patients and negative for gCSC. Among the patients positive for gCSCs, we observed no significant differences in survival between those whose gCSCs were each positive or negative for PDPN, CD133, or nestin. This study strongly supports the prognostic value of gCSCs isolation on the survival of patients with pGBM.

## 1. Introduction

Cancer stem cells (CSCs) are critical in the initiation, maintenance, and progression of tumors and in the development of resistance to therapy [1], although the tumor microenvironment is also important to these processes [2–5]. The accumulation of evidence suggesting a strong association between CSCs and malignancy [6, 7] has led to considerable research on the prognostic role of CSCs in cancer patients [8]. Close associations have been observed between clinical outcomes and the presence of CSC features in various tumors, such as the expression of stem cell markers, genetic features, and the formation of tumor spheres [9–13]. Similar findings have been observed in patients with glioma in that neurosphere formation and the expression of stem cell markers, such as podoplanin (PDPN), CD133, and nestin, were found to be prognostic markers of clinical outcomes [14–16]. In contrast, other studies have found that the presence of CSC features, such as expression of stem cell markers, was not prognostically significant [8, 17, 18], bringing into question the prognostic value of CSC features.

In this study, we assessed the prognostic role of the isolation of glioma CSCs (gCSCs) and expression of stem cell markers (PDPN, CD133, and nestin). These two factors are supposed to be strongly associated with tumor malignancy, in patients with pGBM [15]. We evaluated previously defined populations of gCSCs, with properties that included the ability to form gliomaspheres, to undergo neural differentiation, and to induce tumorigenesis* in vivo* [6]. In this study, a cohort of 39 patients with pGBM was separated into two groups, those positive or negative for gCSCs, and the correlation between gCSC and patient survival was evaluated. We also assessed the expression of stem cell markers (PDPN, CD133, and nestin) in gCSCs and its relationships with patient survival to address the possible prognostic value of these markers in pGBM.

## 2. Materials and Methods

### 2.1. Patient Population

Patients with pGBM treated at two institutions between 2009 and 2013 were included in this study (Table 1); patients with secondary GBM were excluded. All patients were histologically diagnosed by neuropathologists and graded according to the 2007 WHO classification [20]. All patients provided written informed consent, and the study was approved by the Institutional Review Boards of the two institutions (KC10SNSI0466 and 4-2012-0212).

### 2.2. Treatments

All patients received combined therapy, consisting of surgery, followed by concurrent chemotherapy and radiotherapy and adjuvant chemotherapy (Table 2) [19]. The aim of surgery was gross total tumor resection, defined as macroscopic removal of 100% of the tumor mass. Patients not suitable for total resection underwent subtotal resection, defined as removal of <100% but ≥90% of the macroscopic tumor mass, or partial resection, defined as removal of <90% of the macroscopic tumor [21]. The extent of tumor resection was estimated by the neurosurgeons and confirmed by postoperative review of magnetic resonance imaging (MRI) scans. All patients received postoperative adjuvant radiotherapy with concomitant and adjuvant temozolomide (TMZ), as described previously [19]. Recurrent tumors were treated with salvage temozolomide (200 mg/m^2^) [22] in 16 patients and radiotherapy in one patient and not treated in six patients.

### 2.3. Isolation of gCSCs

Tumor specimens had been collected in the operating room from glioblastoma patients undergoing surgery, followed by isolation of gCSCs within 1 hour using a previously described mechanical dissociation method [6]. Only cells that showed the ability to form gliomaspheres, undergo neural differentiation, and induce* in vivo* tumorigenesis, as described in our previous report [6], had been defined as gCSCs. The isolated gCSC preparations had been assayed for the expression of PDPN, CD133, and nestin by immunocytochemistry (Table 1) [6]. These selection procedures and immunocytochemical analyses had been performed using protocols described in our previous report [6]. The survival outcomes of the patients with confirmed pGBM were followed up.

### 2.4. Statistical Analysis

The primary study outcome was overall survival (OS), measured from the date of surgery confirming the diagnosis of pGBM to the date of the last follow-up visit or death. Survival curves were plotted using the Kaplan-Meier method and compared using the log-rank (Mantel-Cox) test. Patients' demographic characteristics were compared using the Mann-Whitney *U* test for continuous variables and by Fisher's exact test for categorical variables. All statistical analyses were performed using SPSS version 18.0KO software (SPSS Korea, Seoul, Korea), with *P* < 0.05 considered statistically significant.

## 3. Results

### 3.1. Patients

Of the 39 pGBM patients treated at our institution from April 2009 to December 2013, fifteen were categorized as positive and twenty-four as negative for gCSCs (Table 1). These patients included 19 males and 20 females, ranging in age from 11 to 82 years. In 15 of the 39 pGBM patients, gCSCs were isolated. Immunocytochemical analysis had showed that [6], of the 15 patients positive for gCSCs, nine had PDPN^+^, fourteen had CD133^+^, and fourteen had nestin^+^ gCSCs (Table 1).

### 3.2. Patient Survival

**Figure 1:**
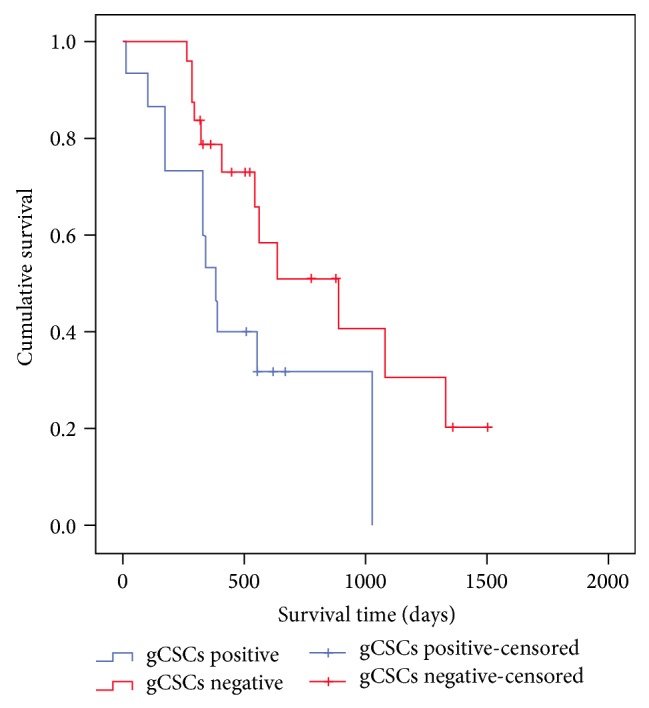
Kaplan-Meier survival curves of pGBM patients positive and negative for gCSCs (*P* = 0.045 as calculated by the log-rank test).

The demographic and clinical characteristics of the 39 pGBM patients are shown in Table 2. The mean survival time of all 39 pGBM patients was 725 days. The mean survival time of the twenty-four gCSC negative patients (855 days) was much longer than that of the 15 gCSC positive patients (520 days). There were no statistically significant differences in age (*P* = 0.188), gender (*P* = 0.653), mean follow-up (*P* = 0.225), extent of surgery (*P* = 0.636), recurrence rate (*P* = 0.192), 1p 19q codeletion (*P* = 0.385), and O-6-methylguanine-DNA methyltransferase (MGMT) methylation (*P* = 1.000) between the gCSC positive and gCSC negative groups of patients. Kaplan-Meier curves displaying the proportion of OS are demonstrated in Figure 1, showing statistically significant difference in cumulative survival (*P* = 0.045) between the two groups.

Among the patients positive for gCSCs, we observed no significant differences in survival between those whose gCSCs were positive (nine patients) or negative (six patients) for PDPN expression (*P* = 0.619), positive (14 patients) or negative (one patient) for CD133 (*P* = 0.079), and positive (14 patients) or negative (one patient) for nestin (*P* = 0.079).

## 4. Discussion

We observed statistically significant difference in OS between gCSC positive and gCSC negative patients with pGBM. As CSCs may influence tumor growth and resistance to treatment [1], gCSCs are supposed to have a prognostic role in predicting the clinical outcome in pGBM patients. CSC features are reported to have been associated with poorer prognosis in various types of cancers [9–13]. Similar findings were reported in patients with a glioma in that the formation of neurosphere correlated with poorer clinical outcomes [14, 23, 24]. Our findings of the statistical analyses present reliable evidence that the isolation of gCSC is an independent prognostic factor for the clinical outcome of patients with pGBM.

To identify further candidate prognostic markers in patients with pGBM, we analyzed the relationship between the presence of PDPN^+^, CD133^+^, and nestin^+^ gCSCs and survival in pGBM patients. Some studies have reported that PDPN, CD133, and nestin expression are prognostic in glioma patients. Ernst et al. [25] and Mishima et al. [15] reported that PDPN expression was prognostic in patients with astrocytomas. Expression of CD133 [26] and nestin [27] was associated with poorer outcomes in many cancers, including brain cancers. In this series, however, there were no statistically significant differences in overall survivals between PDPN^+^, CD133^+^, and nestin^+^ gCSCs and PDPN^−^, CD133^−^, and nestin^−^ gCSCs. The mean survival times of PDPN-expressing gCSCs positive and negative groups were similar, 400 days and 408 days, respectively, and there was no significant difference in OS between two groups (*P* = 0.619). CD133 and nestin were expressed in most of the patients positive for gCSCs and there was only one case of CD133^−^ and nestin^−^ gCSC, so it was impossible to compare the two groups statistically. These findings indicate that the presence of PDPN-, CD133-, and nestin-expressing gCSCs was not prognostic indicators for survival in pGBM patient. This conclusion is supported by data from other studies, which reported that expression of stem cell markers did not have prognostic significance in glioma patients [8, 18, 28].

In this study, only pGBM patients were included for adjusting the grade of glioma. We previously reported that the rates of existence of gCSC increase proportionally as the WHO grades of glioma rise [6]. In the study including samples from various grades of gliomas, the poorer prognosis correlated with the isolation of gCSC should be affected by the grade of glioma because of the close correlation of the gCSC isolation rates and the grade of glioma. Because of the trend of step-by-step increase of gCSC isolation rate according to the WHO grades of gliomas, the stem cell markers should be expressed more in the patients with higher grade gliomas. We showed that most of the gCSCs expressed the stem cell markers, such as CD133 and nestin, in this study.

There are some limitations in this study. The* in vitro* assay for the isolation of gCSC takes length duration and requires the technical expertise to perform it. So, it may not be suitable for predicting the prognosis of the pGBM patients with short survival periods. We investigated the expression of stemness surface antigens only in the neurospheres and not in the parent tumors. Regarding the duration and technical needs of this* in vitro* assay, investigation of the expression of stemness surface antigens in the parent tumors may be more proper for evaluation of the prognosis. These suggest the need for additional studies assessing the associations between the stemness surface antigens in the parent tumor and clinical outcomes in pGBM patients.

## 5. Conclusion

In conclusion, we showed that the presence of gCSCs alone was significantly prognostic of OS in patient with pGBM. Although we found that the presence of PDPN^+^, CD133^+^, and nestin^+^ gCSCs was not prognostic of OS, our findings suggest that this issue warrants further investigation. We are currently performing a continued study in a larger numbers of patients to further address the detailed role of gCSCs in pGBM patients.

## Figures and Tables

**Table 1:** Cohorts of primary glioblastoma patients who were positive or negative for glioma cancer stem cells (gCSCs) [6].

Patients	gCSC candidates	Age (years)	Sex	Podoplanin	CD133	Nestin
gCSC positive	gCSC0315	57	F	−	+	+
	gCSC0426	44	F	−	+	+
	gCSC0520	72	F	−	+	+
	gCSC0713	55	F	−	−	−
	gCSC0504	39	M	+	+	+
	gCSC1120	48	F	+	+	+
	gCSC0503	64	M	+	+	+
	gCSC0114	53	M	−	+	+
	gCSC0213	51	F	−	+	+
	gCSC0228	68	M	+	+	+
	gCSC0308	61	M	+	+	+
	gCSC0924	11	M	+	+	+
	gCSC0510	49	F	+	+	+
	gCSC0627	61	M	+	+	+
	gCSC0520	34	M	+	+	+

gCSC negative	gCSC0406	38	F			
	gCSC08241	71	F			
	gCSC1005	59	F			
	gCSC1124	63	F			
	gCSC0226	28	M			
	gCSC0309	59	M			
	gCSC0803	60	F			
	gCSC08242	65	F			
	gCSC0620	66	F			
	gCSC0928	46	M			
	gCSC1108	82	M			
	gCSC0219	60	M			
	gCSC0528	66	F			
	gCSC0610	49	M			
	gCSC0702	63	M			
	gCSC0709	24	M			
	gCSC0816	57	M			
	gCSC0822	62	F			
	gCSC1118	57	F			
	gCSC1218	44	M			
	gCSC0102	68	M			
	gCSC0106	57	F			
	gCSC0529	60	F			
	gCSC1102	57	M			

gCSC: glioma cancer stem cell; M: male; F: female; +: positive for expression as assessed using immunocytochemical methods [6]; −: negative for expression, as assessed using immunocytochemical methods [6].

**Table 2:** Demographic characteristics of the pGBM patient.

Characteristics	gCSCs positive	gCSCs negative	*P* value^*^
	(*N* = 15)	(*N* = 24)	
Age (years)			0.188
Median	53 ± 15	60 ± 13	
Range	11–72	28–82	
Sex (number [%])			0.653
Male	8 (53%)	11 (46%)	
Female	7 (47%)	13 (54%)	
Mean follow-up (months)	13.5 ± 8.4	19.9 ± 12.4	0.225
Pathological diagnosis	Primary glioblastoma	Primary glioblastoma	
Treatment	Surgery + Stupp's regimen [19]	Surgery + Stupp's regimen [19]	
Extent of surgery (number [%])			
Total resection	10 (67%)	14 (58%)	0.636
Subtotal resection	4 (27%)	8 (33%)	
Partial resection	1 (7%)	2 (8%)	
Recurrences (number [%])	11 (73%)	12 (50%)	0.192
Treatment of recurrences (number [%])			
Radiation therapy	1 (9%)	0 (0%)	
Adjuvant temozolomide	6 (55%)	10 (83%)	
None	4 (36%)	2 (17%)	
1p 19q codeletion by FISH	1 (7%)	0 (0%)	0.385
MGMT methylation by PCR	7 (47%)	11 (45%)	1.000

pGBM: primary glioblastoma; gCSCs: glioma cancer stem cells; MGMT: O-6-methylguanine-DNA methyltransferase. ^*^By Mann-Whitney test for continuous variables and by Fisher's exact test for categorical variables.
